# Massive pulmonary embolism secondary to longstanding traumatic femoropopliteal pseudoaneurysm associated with arteriovenous fistula

**DOI:** 10.1186/1749-8090-9-39

**Published:** 2014-02-22

**Authors:** Ahmad K Darwazah, Mohammed Eida, Hamad Madi, Issa Khdour, Naser Hanbali, Hasan Ismail

**Affiliations:** 1 Department of Cardiac Surgery, Ramallah and Makassed Hospital, Mount of olives, 91194 Jerusalem, Israel; 2 Anaesthesia Ramallah Hospital, West Bank, Israel

**Keywords:** Pulmonary embolism, Trauma, Bullet injury, Femoropopliteal pseudoaneurysm, Arteriovenous fistula

## Abstract

Pulmonary embolism is a common clinical condition associated with high mortality.

The majority of pulmonary emboli originate from deep venous thrombosis in the popliteal and femoral veins.

We present a rare case of a 21-year-old caucasian male patient with massive pulmonary embolism. The source of emboli originated from thrombosed femoropopliteal pseudoaneurysm associated with arteriovenous fistula which was caused by a bullet injury 7 years before. The patient underwent successful surgical pulmonary embolectomy followed by aneurysmectomy with reconstruction of femoral and popliteal vessels.

## Background

Massive PE is a serious fatal condition with an estimated incidence of 4.5% [1]. It is characterized by hemodynamic instability and occlusion of more than 50% of pulmonary vasculature [2].

Various therapeutic options are available including anticoagulation, thrombolysis, catheter embolectomy and surgery.

We report a young patient with massive PE associated with peripheral pseudoaneurysm. Both conditions were treated successfully by surgical intervention.

## Case presentation

A 21-year-old caucasian male patient was referred as an emergency case of massive pulmonary embolism.

Two days before admission, the patient had epigastric pain and vomiting. He was diagnosed as having gastritis and was treated accordingly.

Twelve hours after observation, his condition deteriorated due to chest pain and tachypnea. Blood pressure was 90/60 mmHg with regular pulse of 120. Respiratory rate was 22 breath/ min. with oxygen saturation of 80% at room air.

Electrocardiography showed sinus tachycardia, large S wave in lead I, inverted T wave in Lead III and incomplete right BBB. Cardiac enzymes, troponin, and D-dimer were all negative.

Acute pulmonary embolism was suspected despite the fact that D-dimer was negative and the patient had no past history of DVT or recent operation or trauma. However, the patient gave a history of a gunshot to the right thigh when he was 14 years old.

Echocardiography showed severe dilatation of the right ventricle, atrium and pulmonary artery with no evidence of emboli. Tricuspid valve was severely incompetent with PAP of 55 mmHg.

**Figure 1:**
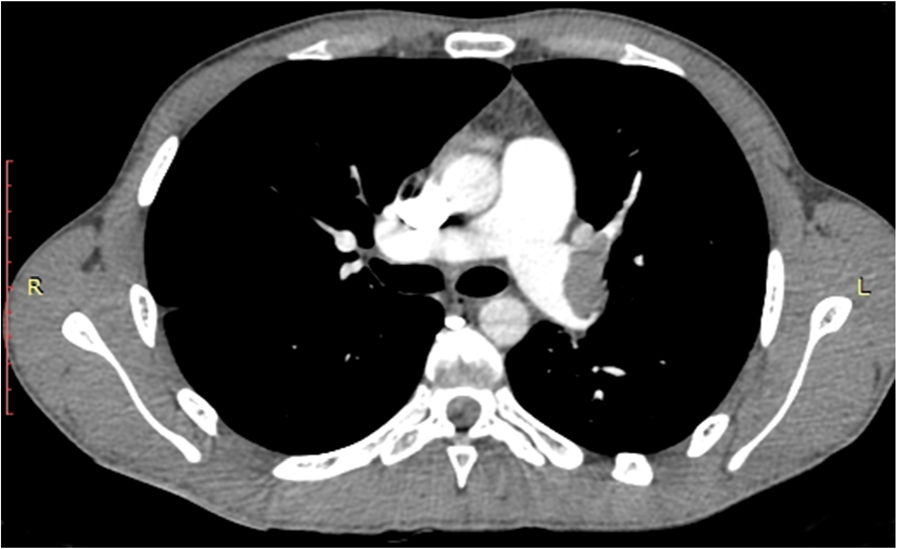
Preopoperative CT angiography showing massive LPA embolism with small emboli in the right upper branch of RPA.

CT angiography showed a massive left PA embolism with small emboli in the right upper branch of the right PA (Figure 1). Heparin infusion was commenced and the patient was transferred.

On arrival, the patient was immediately transferred to the operating room due to hemodynamic instability (BP 60/40 mmHg, pulse 140, CVP 22 mmHg and high respiratory rate).

**Figure 2:**
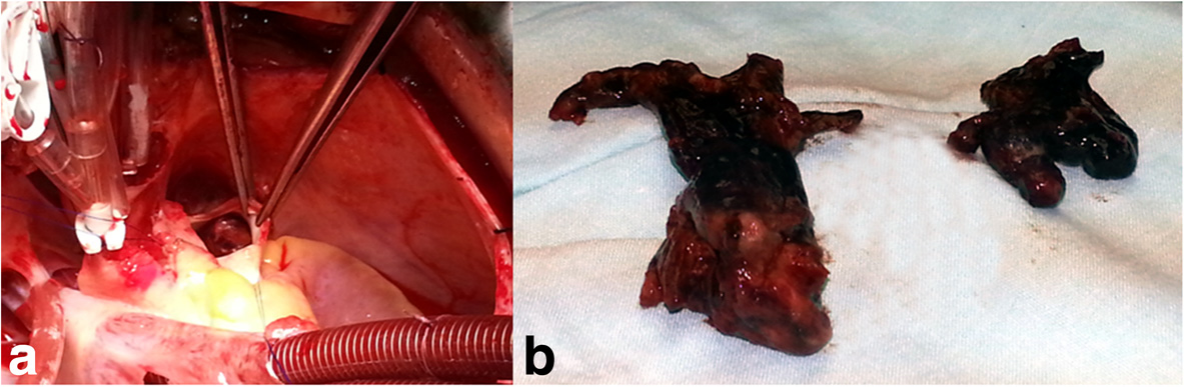
Intraoperative longitudinal incision of the main PA showing (a) Embolus in the left PA (b) Embolus after extraction.

The heart was exposed through median sternotomy.The procedure was performed under cardioplegic arrest and mild hypothermia. Two huge emboli from left PA and 3 small emboli from right PA were extracted through a longitudinal incision in the main PA extending to the left branch (Figure 2a,b). No emboli or PFO were seen in the right atrium and ventricle. Weaning from bypass was uneventful.

The patient remained hemodynamically stable postoperatively receiving minimal inotropic support and heparin infusion.

**Figure 3:**
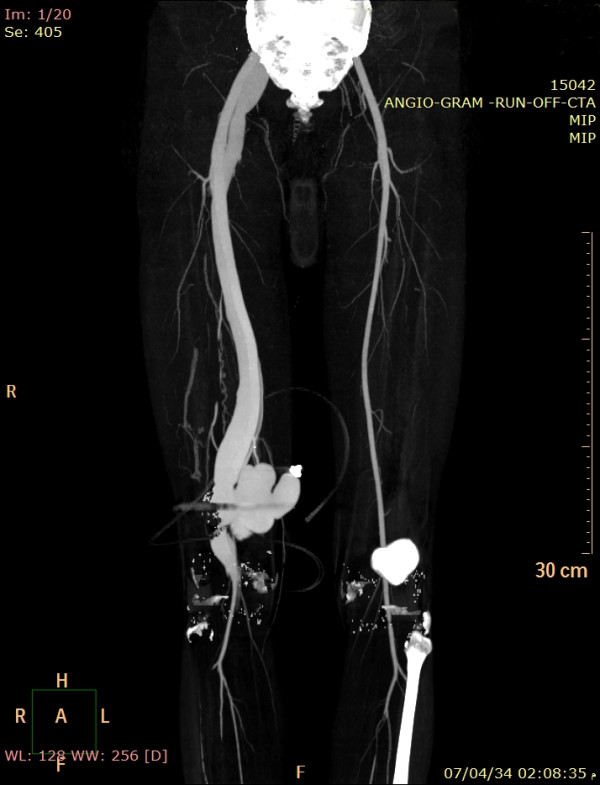
**CT angiography showing a Femoropopliteal Pseudoaneurysm with dilated deep veins.** Bullet shrapnels seen around pseudoaneurysm.

On the 3^rd^ postoperative day, the site of previous gunshot was evaluated by duplex ultrasound and CT angiography which revealed a femoropopliteal peudoaneurysm associated with arteriovenous fistula (Figure 3). Both inferior vena cava and the deep venous system were patent with no evidence of DVT.

**Figure 4:**
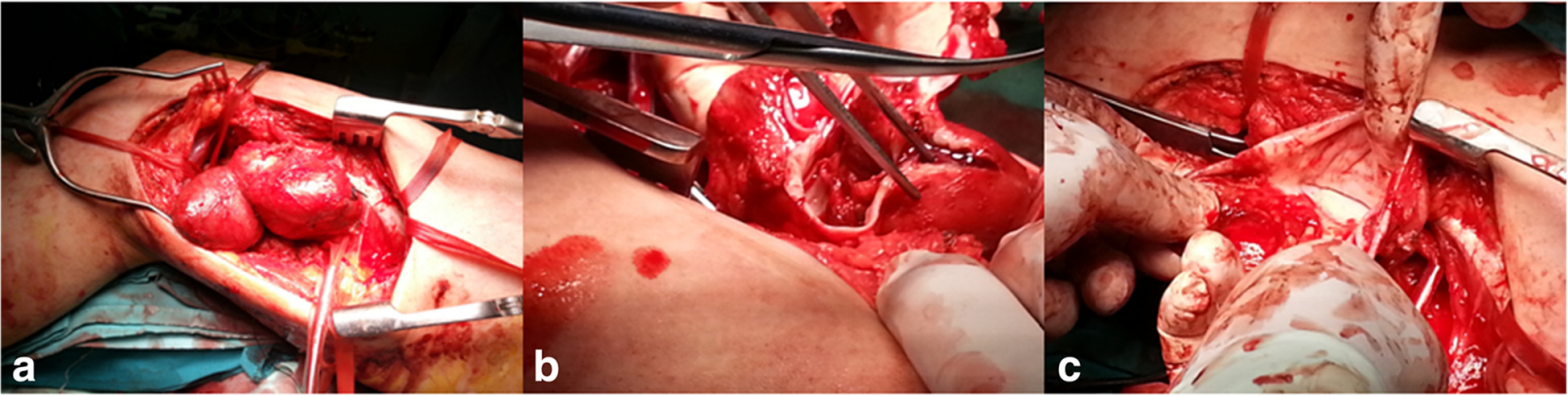
Intraoperative exposure of the thigh showing (a) multilobulated pseudoaneurysm (b) organized thrombus (c) A-V fistula.

Through a midline incision at the lower end of the thigh, femoral and popliteal vessels were dissected. A multilobulated thrombosed pseudoaneurysm 12×10 cm with fistula was found (Figure 4a,b,c). The femoral vein was separated and repaired. Femoral artery was reconstructed after excision of the pseudoaneurysmal sac and anastomosed to the distal end of the popliteal artery. Both operative and postoperative recovery were uneventful.

**Figure 5:**
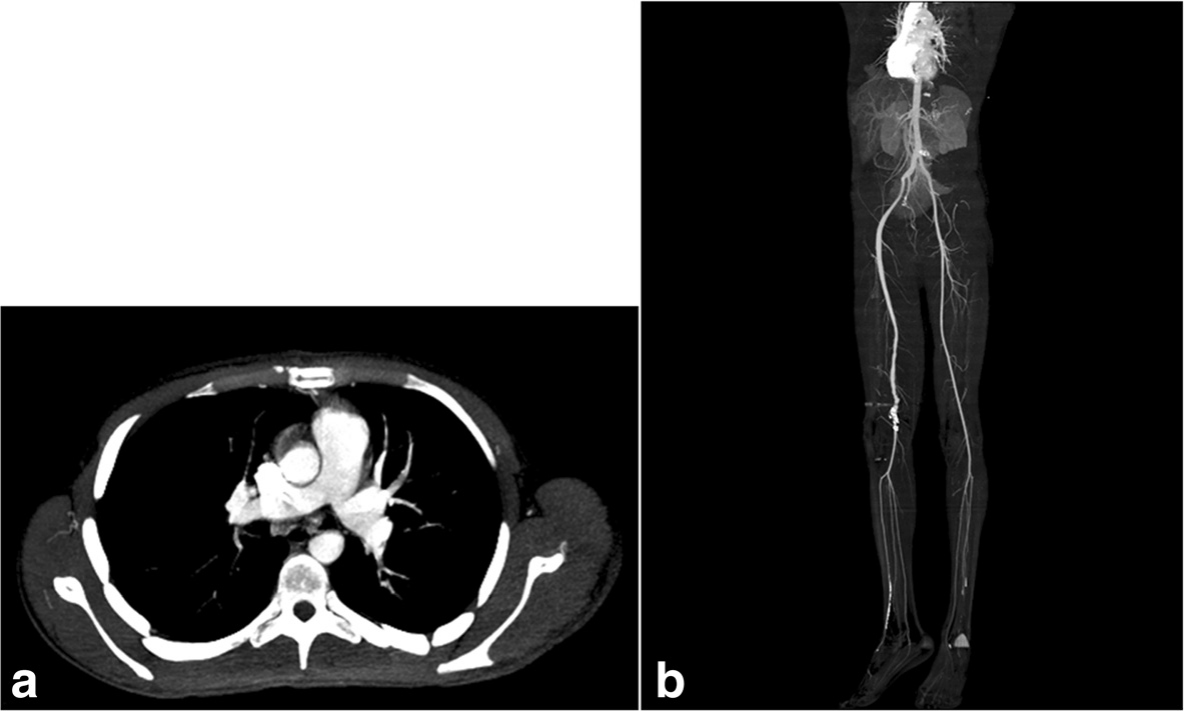
Postoperative CT angiography (a) showing a clear pulmonary arteries (b) patent peripheral arteries.

Postoperative CT angiography showed clear pulmonary and patent peripheral arteries with good distal flow (Figure 5a,b).

The patient was discharged home after two weeks receiving aspirin and marivan.

## Discussion

Pulmonary embolism (PE) is a common clinical condition with an estimated incidence of 2.3 per 10,000 [3].

Despite advances in diagnosis and therapy, it is still associated with high mortality. In severe cases with massive PE mortality rate may reach up to 52% [4].

The main therapy of PE is anticoagulation using heparin. However, in severe cases of massive or submassive occlusion of PA such treatment is insufficient.

Thrombolytic therapy is considered to be the first line of treatment of PE [5]. Thrombolysis causes rapid resolution of pulmonary emboli and subsequent restoration of hemodynamic stability, and right ventricular function [3].

Despite these facts, thrombolysis can be associated with severe bleeding, intracranial hemorrhage and is unsuccessful in 5-10% of patients [3,6].

Minimal invasive technique using catheter embolectomy is effective in removal of clots and recovery of right ventricular function. However, it is associated with recurrence of pulmonary emboli, pulmonary hypertension, hemorrhage, injury and perforation of pulmonary arteries [4].

Surgical pulmonary embolectomy has been traditionally reserved as a last resort of treatment. It is commonly employed in patients with massive PE with cardiogenic shock or among those with failed medical therapy. The early results were disappointing due to high mortality rate reaching 60% [7].

Recently, the concept of using surgery has been changed. Its indications expanded to include more stable patients. The rationale behind such change was based on the importance of right ventricular function in determining early and late survival. The mortality rate of patients with RV failure undergoing surgery may reach 30%, while patients with cardiogenic shock or previous preoperative cardiac arrest the mortality increases to 70% [7].

Various studies showed a significant reduction of mortality between 3.8-6% when surgical intervention was performed early among unstable patients with massive PE or in stable patients with submassive PE with RV dysfunction [4,7].

The precise diagnosis of PE in our patient was obtained by CT angiography. Although, transthoracic echocardiography failed to visualize PA emboli, the data obtained regarding RV dilatation, PA dilatation, pulmonary hypertension and severe tricuspid regurgitation were important to suspect PE and subsequently to commence early treatment and to perform further investigations.

Our decision to perform early surgery was based on the previous observation of the above studies. Our patient had a massive left PA embolism, his hemodynamic parameters drastically changed on arrival and there was evidence of RV dysfunction on echocardiography.

Although the current guidelines recommended the use of thrombolytic agents in massive PE, such modality of treatment was not used primarily due to hemodynamic instability of the patient, moreover the outcome of surgery after possible failure of thrombolytic therapy is associated with high mortality.

The use of inferior vena cava filters after pulmonary embolectomy is debatable. Various studies [7,8], recommended its routine use to prevent recurrent pulmonary emboli. While, others [9] have demonstrated no evidence of recurrent emboli in absence of these filters. In the present case, IVC filters were not used as the patient was already anticoagulated and the source of PE originated from the thrombosed pseudoaneurysm and not from the deep venous system.

Among the previously operated patients with PE, the risks of developing embolisation was related to recent trauma or surgery, malignancy, hypercoagulable conditions, immobility, pregnancy and the presence of deep vein thrombosis [2]. In 20% of cases, no obvious risk factor could be detected [1].

The risk factor for the development of PE in our patient was rather interesting. The source of emboli came from a thrombosed femoropopliteal pseudoaneurysm which was associated with fistulous connection with the femoral vein caused by a bullet injury. The initial vascular trauma was clinically unrecognised and presented several years later.

The thrombotic material traversed across the A-V fistula to reach the pulmonary artery instead of showering distally. This unusual route was facilitated by the associated injury of the popliteal artery at the initial trauma. As seen by preoperative CT angiography, the distal end of the injured popliteal artery was extremely narrowed. This prevented distal embolisation and created more pressure inside the pseudoaneurysm, thus forcing emboli across the large fistula to reach pulmonary artery.

Both A-V fistula and pseudoaneurysm formation are well recognized complications of vascular trauma. Early recognition and repair can avoid such complications. Longstanding A-V fistula causes irreversible degenerative changes in the arterial wall causing pseudoaneurysm formation and thrombosis, dilatation of the involved veins, congestive heart failure, limb oedema and ischemia. The development of pseudoaneurysm may appear years after trauma and can develop with or without repair of traumatic A-V fistula [6,10]. These patients often present with peripheral arterial occlusion and intermittent claudication [10].

Treatment of traumatic pseudoaneurysm with A-V fistula is surgical resection and primary repair. In neglected cases, surgery is often difficult and associated with high incidence of bleeding. Under such circumstances, stent graft implantation is an effective alternative technique [11].

Our decision to perform surgery was due to the presence of thrombosis inside the aneurysm, extension of the aneurysm to involve the popliteal artery with narrowing of its distal end and the young age of the patient.

Surgical excision and repair was not easy due to the presence of extensive adhesions and congested vessels. However, meticulous technique was sufficient to obtain the best outcome.

## Conclusions

Massive PE may rarely occur secondary to thrombosed femoropopliteal psuedoaneurysm associated with fistula. The early diagnosis and quick decision to perform surgery is associated with a high success rate.

## Consent

Written informed consent was obtained from the patient for publication of this case report and any accompanying images. A copy of the written consent is available for review by the Editor-in-Chief of this journal.

## Competing interests

The authors declare that they have no competing interests.

## Authors’ contributions

All authors were involved in the management of the patient. AKD carried out the operation and writing the manuscript. ME, HM and IK assisted during surgery and follow up of the patient. NH and HI were involved in anaesthesia and postoperative management of the patient. All authors read and approved the final manuscript.
